# A randomized, blinded, controlled trial to assess sand fly mortality of fluralaner administered orally in dogs

**DOI:** 10.1186/s13071-018-3231-8

**Published:** 2018-12-05

**Authors:** Sonia Ares Gomez, Javier Lucientes, Juan Antonio Castillo, Maria Paz Peris, Sarah Delacour, Paula Ortega, Ronald-Vladimir Oropeza, Albert Picado

**Affiliations:** 1 ISGlobal, Hospital Clínic - Universitat de Barcelona, Barcelona, Spain; 2 Department of Animal Pathology, Faculty of Veterinary Medicine at the University of Zaragoza, Zaragoza, Spain

**Keywords:** Zoonotic visceral leishmaniasis (ZVL), Sand flies, Dogs, Systemic insecticides, Fluralaner

## Abstract

**Background:**

*Leishmania infantum* is the parasite responsible for the disease in humans known as zoonotic visceral leishmaniasis (ZVL). Dogs are considered the main domestic reservoir of ZVL and sand flies are the proven vectors. The use of systemic insecticides in dogs has been studied as an alternative strategy to control ZVL in endemic areas. One systemic insecticide in dogs, fluralaner, has a proven anti-sand fly effect in membrane-fed studies. However, the efficacy and duration on sand flies directly feeding from dogs treated with fluralaner remains unknown.

**Methods:**

Direct feeding bioassays were performed on 10 beagle dogs that had been randomly assigned to two groups: one with five dogs orally treated with Bravecto® (fluralaner) and other five as a control. About 30 females of *Phlebotomus papatasi* were allowed to directly feed from dogs at seven days before the administration of the treatment and Days 3, 17, 31, 45 and 73 post-treatment. Sand fly mortality after feeding was observed every 24 h for 5 days. The Kaplan-Meyer method, Henderson-Tilton formula and a negative binomial mixed model were used to respectively calculate: (i) mortality and its 95% confidence interval (CI); (ii) efficacy of the insecticide at killing sand flies in 24 h; and (iii) differences in the risk of sand fly death at 24 h after feeding.

**Results:**

Control sand fly mortality 24 h after feeding was always ≤ 20% and mortality in the fluralaner group ranged from 2% (95% CI: 0–4%) 7 days before treatment to 100% at 3 days post-treatment. Fluralaner efficacy was 100, 93, 94 and 75% at Days 3, 17, 31 and 45, respectively (*P* < 0.0001). The increase in the risk of sand fly death was 32.9 (95% CI: 4–263), 76 (95% CI: 8–705), 95.8 (95% CI: 9–1029) and 10.6 times (95% CI: 1.43–79) on Days 3, 17, 31 and 45, respectively

**Conclusions:**

The efficacy of fluralaner, orally administered to dogs, against sand-flies was above 90% for 31 days. Fluralaner administered to dogs should be further evaluated as a control strategy in ZVL endemic areas.

**Electronic supplementary material:**

The online version of this article (10.1186/s13071-018-3231-8) contains supplementary material, which is available to authorized users.

## Background

Fluralaner is a systemic insecticide from the ixosazoline group used for the control of ectoparasites in companion animals. Bravecto® is the commercialized name of flavored chewable tablets of fluralaner (25 mg/kg body weight) registered for dogs to control fleas and ticks infestations for 12 weeks [1]. Fluralaner belongs to the isoxazoline group. Isoxazolines act at the central nervous system or the neuromuscular junction of the insect blocking GABA-gated chloride channels, thus disrupting neuronal signaling and muscle regulation causing insect death [2, 3]. Based on the mechanism of action, fluralaner and other systemic insecticides used in dogs may have an anti-sand fly effect [4, 5]. The effect of fluralaner on sand fly mortality was demonstrated in a randomized clinical trial in dogs using membrane feeding bioassays [6]. This proof of concept study reported sand fly mortalities between 60–80% for 30 days in dogs treated with fluralaner [6]. However, the high sand fly mortality in the control group precluded accurately estimating the mortality caused by fluralaner. Membrane-feeding was also used to demonstrate that fluralaner, mixed with rabbit blood, was effective against different species of sand flies [5].

Some species of sand flies are the vectors of *Leishmania infantum*, the parasite causing zoonotic visceral leishmaniasis (ZVL). ZVL is a major public health concern in some countries and regions (e.g. 3000 cases with 10–19% lethality in Brazil per year [7]). Dogs, which are also affected by the parasite, are the main reservoir and they are often targeted by the leishmaniasis control programs in endemic countries. In Brazil, *L. infantum* infected dogs are culled to reduce the source of infection [7]. This strategy is controversial and has had a limited impact on the number of ZVL cases in humans [8–10]. The use of insecticide-impregnated collars in dogs [11–13] has been evaluated as an alternative to dog culling. Community-wide use of insecticide-impregnated collars has been proven to reduce the risk of *L. infantum* infection in humans [13, 14] but its use at a regional or national level has been limited due to several factors, namely the price of collars, collar losses, collar removal by dogs owners and high percentage of unreachable stray dogs [12, 14, 15]. Other topical insecticides in dogs such as topical lotions of deltamethrin, permethrin and fenthion, have also been suggested in vector control [16, 17]. The use of systemic insecticides in dogs has been proposed as a vector control tool in endemic regions. A modelling study showed that treating 80% of dogs with a systemic insecticide that induces a sand fly mortality over 65% for 7.4 months would reduce the risk of *L. infantum* infection in humans by 80% [18].

To the best of our knowledge, there are currently no systemic insecticides for dogs registered against sand flies. Fluralaner, which can be given orally, is registered for dogs against ticks and fleas and has a proven anti-sand fly effect [5], could be a good control tool for ZVL in endemic areas [4, 18] In fact, Miglianico et al. [5] suggested the repurposing of fluralaner so it can be used in humans to control leishmaniasis and other vector-borne diseases. To further evaluate the efficacy and duration of fluralaner administered to dogs against sand flies we performed a blinded, randomized clinical trial using direct-feeding bioassays.

## Methods

### Study site and dogs

A total of 10 beagles (5 males and 5 females) between 12 and 24 months-old and weighting between 11–13 kg were used. The dogs were obtained from a licensed vendor and acclimatized to the study facility for more than 40 days prior to the beginning of the study. Dogs were uniquely identified by a subcutaneous microchip. The dogs were housed in individual inside-outside kennels that conformed to accepted guidelines for animal welfare. Dogs were fed a commercial dry dog ration once a day, water was provided *ad libitum*, and received regular exercise and social interaction. At the beginning of the study all dogs were clinically healthy and not clinically pregnant as determined by a veterinarian. Dogs had not been treated with drugs, baths, shampoos, or pesticides within 4 weeks preceding the beginning of the study and were not treated during the course of the study other than with fluralaner in the treated group.

### Study design

The study was designed to detect at least 65% insecticide efficacy of fluralaner on blood-fed *Phlebotomus papatasi* feeding on dogs, with a power of 0.8 and an alpha of 0.05.

Two groups of 5 dogs were randomly selected blocking by gender. Once two groups were formed, one with 3 females and 2 males and other with 2 females and 3 males, treatment was randomly assigned to one of the groups. The random selection was done using the sample function in R [19].

At Day 0, the dogs in the treatment group received one chewable tablet for medium-sized dogs (> 10 to 20 kg) of Bravecto®, equivalent to 500 mg of fluralaner, at the time of feeding, as indicated in the label. Control dogs received their regular ration only.

The effect of Bravecto® on sand flies was assessed using direct-feeding bioassays at Days 3, 17, 31, 45 and 73. Additionally, a direct feeding bioassay was performed 7 days before the administration of the treatment (Day -7).

*Phlebotomus papatasi* females, two- to seven-days-old from a colony reared in the University of Zaragoza were used in the direct-feeding bioassays. The colony was established in 2012 with *P. papatasi* trapped in the province of Zaragoza (Spain). The colony was maintained at 27 °C, 80% relative humidity and photoperiod of 17:7 h (light:dark) [20].

**Fig. 1:**
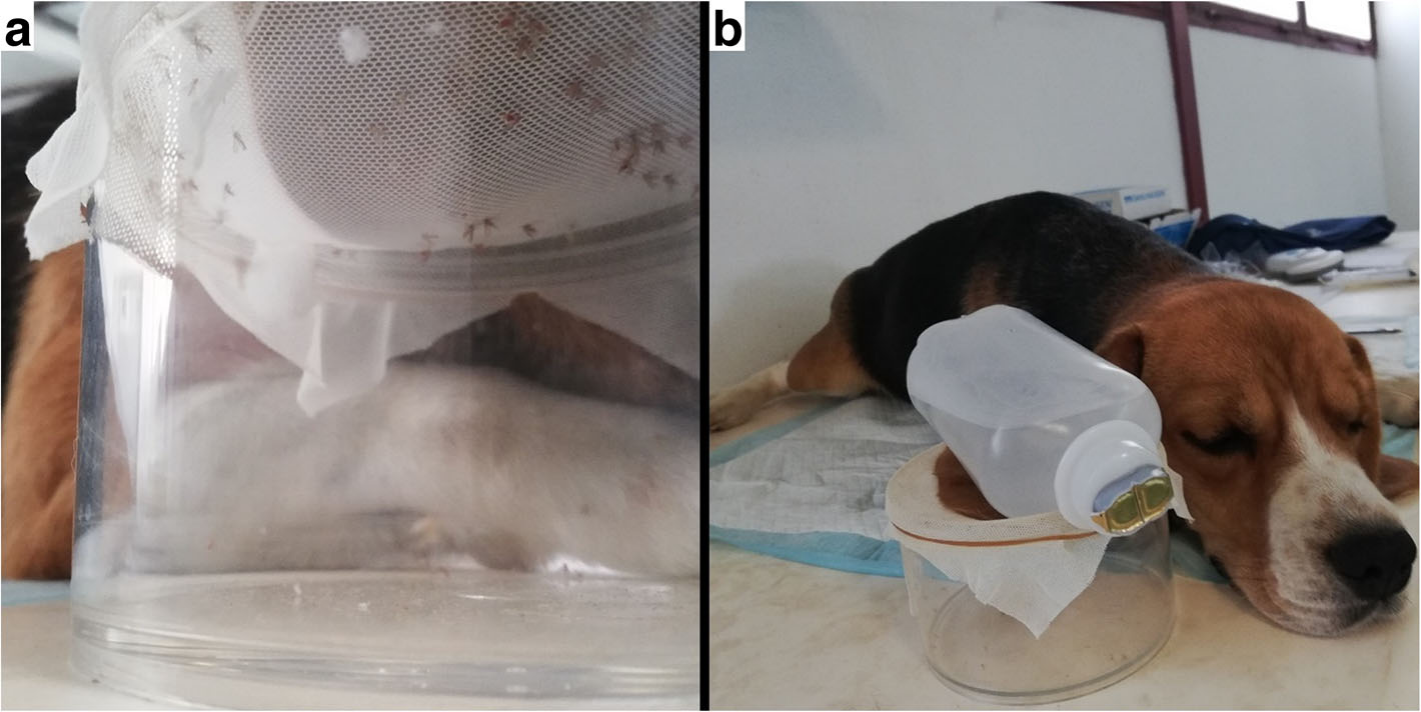
Direct feeding bioassay of *Phlebotomus papatasi* directly feeding on a sedated dog. **a** Detail of the inner part of the ear exposed to the bite of about 30 *P. papatasi* females located inside a round container covered by a cotton net that allowed the sand flies to feed through. **b** Sedated dog with the ear exposed to sand flies being warmed by a bottle of 250 ml of normal saline previously warm to 37 °C

On the days of the bioassay, fasted dogs were sedated with 0.5 mg/kg of intramuscular dexmedetomidine (Dexdomitor®). Once the dog was sedated, the previously shaved inner part of the ear was exposed to the bite of about 30 *P. papatasi* females. To facilitate the feeding and the recovery of blood-fed sand flies during the direct feeding bioassays [21, 22] the sand flies were introduced in a round container (11.5 cm diameter and 7.5 cm height) covered by a cotton net that allowed them to feed through (Fig. 1a). To keep the ear warm, one bottle of 250 ml of normal saline previously warm to 37 °C was placed on top (Fig. 1b). One hour or at least 30 min later, when most of the females were engorged, the sand flies were removed. The dogs received one intramuscular injection of atipamezole (0.25 mg/kg, Antisedan®) to revert the anesthesia.

For the ease of observation, engorged sand flies were separated in groups of five into a plastic cup of 90 ml and 60 mm diameter with sucrose solution provided daily. The plastic cups were introduced into an incubator where humidity was controlled. Sand fly mortality was observed every 24 h during five days. Sand fly mortality and its 95% CI was calculated at each day for 5 days post-feeding using the Kaplan-Meyer method [23].

The researchers manipulating the sand flies and observing sand fly mortality were blind to treatment allocation.

### Statistical analysis

Two methods were used to assess the sand fly mortality due to fluralaner. The Henderson-Tilton formula was used to estimate the efficacy of the insecticide at killing sand flies [24], and a negative binomial mixed model was used to estimate the risk of sand fly death due to fluralaner over time [25]. Both analyses used the sand fly mortality observed 24 h post-ingestion as the outcome.

Efficacy was measured as the percentage of sand fly mortality due to the insecticide effect and was calculated according to the following adapted Henderson-Tilton formula [24, 26]:

$$ \mathrm{Eficcacy}\kern0.5em \left(\%\right)=\kern0.5em \left(1-\frac{\mathrm{No}.\kern0.5em \mathrm{of}\kern0.5em \mathrm{engorged}\kern0.5em \mathrm{SF}\kern0.5em \mathrm{in}\kern0.5em \mathrm{Co}\kern0.5em \times \kern0.5em \mathrm{No}.\kern0.5em \mathrm{of}\kern0.5em \mathrm{SF}\kern0.5em \mathrm{alive}\kern0.5em \mathrm{in}\kern0.5em \mathrm{T}\kern0.5em \mathrm{after}\kern0.5em 24\kern0.5em \mathrm{h}}{\mathrm{No}.\kern0.5em \mathrm{of}\kern0.5em \mathrm{SF}\kern0.5em \mathrm{alive}\kern0.5em \mathrm{in}\kern0.5em \mathrm{Co}\kern0.5em \mathrm{after}\kern0.5em 24\kern0.5em \mathrm{h}\kern1em \times \kern0.5em \mathrm{No}.\kern0.5em \mathrm{of}\kern0.5em \mathrm{engorged}\kern0.5em \mathrm{SF}\kern0.5em \mathrm{in}\kern0.5em \mathrm{T}\kern0.5em }\right)\kern0.5em \times \kern0.5em 100 $$

where SF is sand flies, Co is control group and T is treatment group. Fisher’s exact test was used for each experimental day to test if differences in sand fly mortality were significantly different at alpha 0.05. The European Medicine Agency uses as reference method a similar formula and efficacies above 80% should be achieved to demonstrate efficacy of ectoparasiticides [27].

In negative binomial mixed model the variable that identified each individual dog was included as random effect [25]. The model explanatory variables were treatment, day of study, and the interaction between treatment and Day. The interaction term allowed us to estimate differences in the sand fly mortality across time. The model dependent variable was the log of the number of sand flies dead 24 h post-feeding given the total number of engorged sand flies (offset). The exponential of the model estimates represents the incidence rate ratios (IRR) which indicate how many times the risk of death increases due to fluralaner.

All analyses were conducted in R (version 3.3) [19]. The *MASS* package [28] was used to conduct the negative binomial regression. The *survival* and *coxme* packages [29] were used to conduct the Kaplan-Meier table.

## Results

The study was conducted between May and July 2018. No adverse effects related to the treatment were observed in any animal during the course of the study. A total of 909 and 849 specimens of *P. papatasi* were used in the treatment and control groups, respectively (Table 1). Control sand fly mortality 24 h after feeding was always ≤ 20% as established in the EU guidelines [27]. Kaplan-Meyer mortality at 24 h post-feeding (%, and 95% CI) per group and bioassay are reported in Table 1; the data at 48, 72, 96 and 120 h post-feeding are provided in Additional file 1: Table S1 and the data for 24 h sand fly mortality per dog are provided in Additional file 1: Table S2. Sand fly mortality 24 h after feeding in the control group ranged from 1% (95% CI: 0–3%) to 20% (95% CI: 13–28%) corresponding to Days 31 and 73 respectively. Mortality in the fluralaner group ranged from 2% (95% CI: 0–4%) at Day -7 (before treatment) to 100% at Day 3 after treatment. At Days 17 and 31 the 24 h mortality and its 95% CI were above 90%. A decrease to 77% (95% CI: 72–82%) was observed at Day 45 and a further drop to 42% (95% CI: 35–49%) at Day 73 (Table 1).

**Table 1:** Observed efficacy and induced mortality of fluralaner against sand flies 24 hours after exposure on dogs

Direct feeding	Control			Fluralaner				
	*n* ^a^	Deaths^b^*n* (%)	95% CI	*N* ^a^	Deaths^b^*n* (%)	95% CI	Efficacy^c^(%)	*P*-value^d^
Day -7	138	3 (2)	0–5	123	2 (2)	0–4	0	0.75
Day 3	147	6 (4)	0–7	151	151 (100)	na^e^	100	<0.0001
Day 17	150	3 (2)	0–4	146	143 (98)	96–100	93	<0.0001
Day 31	148	2 (1)	0–3	159	150 (95)	91–98	94	<0.0001
Day 45	141	13 (10)	5–15	148	138 (89)	84–93	75	<0.0001
Day 73	153	34 (22)	13–28	182	75 (42)	35–49	26	<0.08

^a^Number of sand flies exposed to direct-feed on dogs

^b^Number (%) of sand flies dead 24 h after direct-feeding

^c^Efficacy as the percentage of phlebotomine mortality due to the insecticide effect calculated according to adapted Henderson-Tilton’s formula

^d^(Two-sided) probability values associated with the comparison on sand fly mortality between control and fluralaner using Fisher’s exact test

^e^All samples showed 100% death so there is no 95% CI

**Fig. 2:**
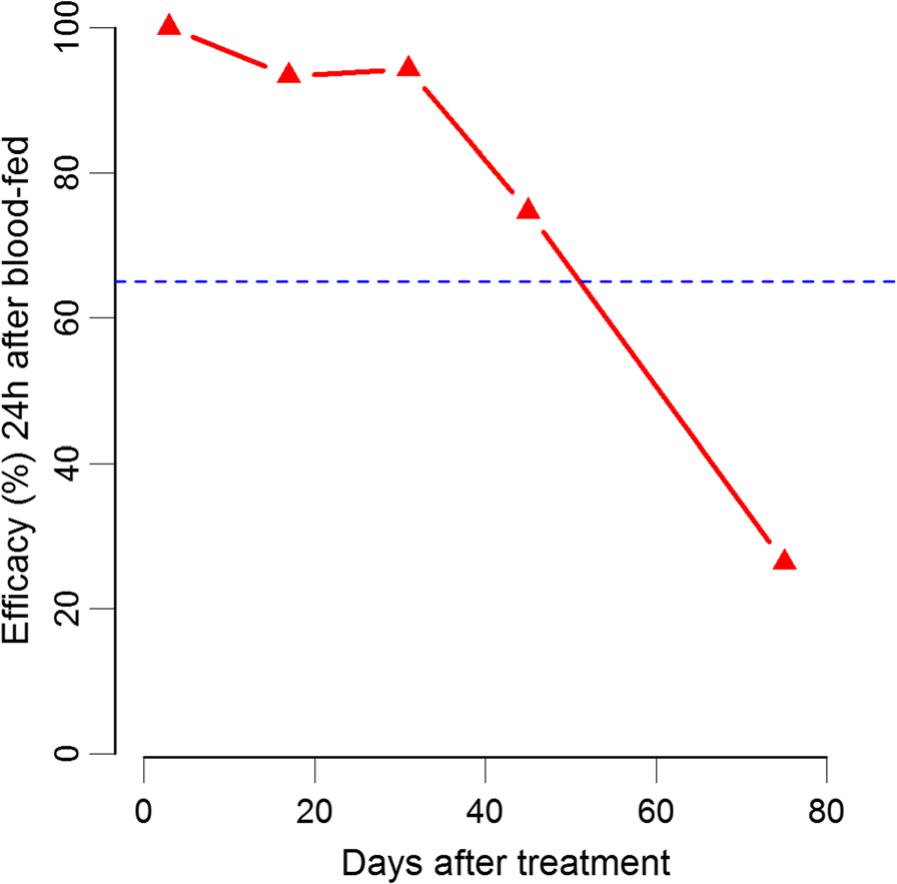
Corrected efficacy (%) according to Henderson-Tilton’s formula for *Phlebotomus papatasi* 24 h after feeding on dogs treated with one chewable tablet of Bravecto® on Days 3, 17, 31, 45 and 73 after treatment. The interrupted line indicates the efficacy threshold of 65%

Fluralaner showed efficacies of 100, 93 and 94% at Days 3, 17 and 31, respectively (*P* < 0.0001; Table 1). Between Days 45 (efficacy 75%; *P* < 0.0001) and 73 (29 %; *P* = 0.08) the efficacy dropped below the threshold of 65% (Fig. 2).

**Fig. 3:**
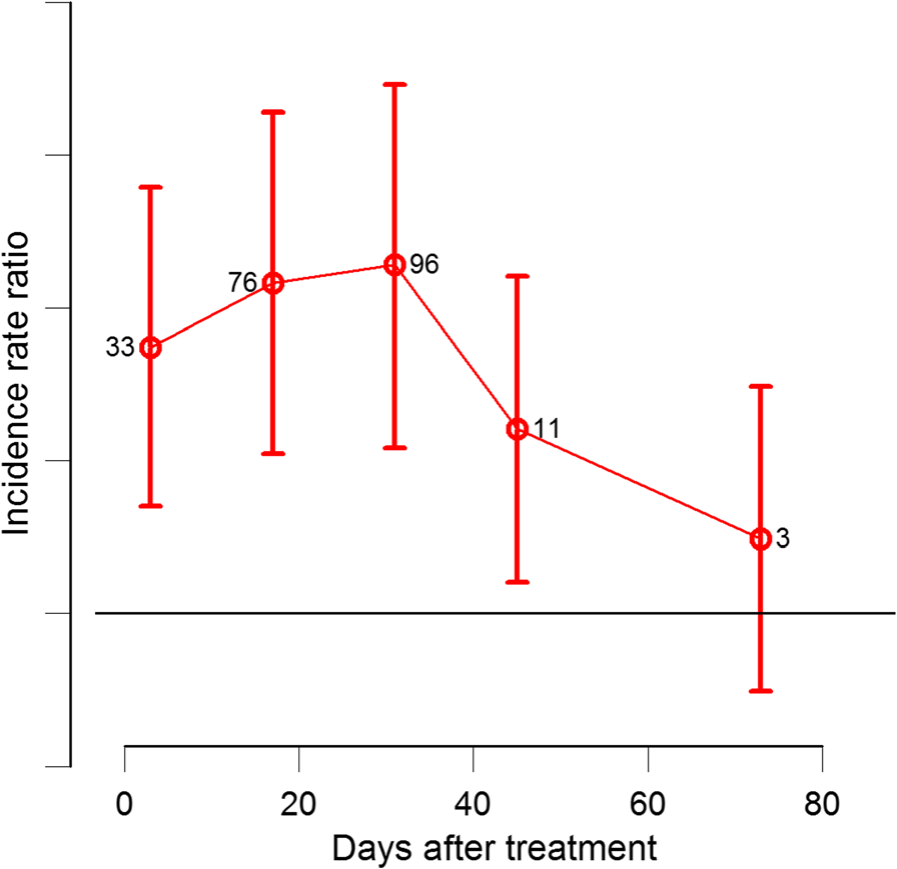
Incidence rate ratios (IRR) for mortality of *Phlebotomus papatasi* 24 h after feeding on dogs treated with one chewable tablet of Bravecto® compared with control on Days 3, 17, 31, 45 and 73 after treatment. IRR was estimated from mixed negative binomial model were variable dog was included as a random effect, treatment, days after treatment, and their interaction were explanatory variables. The horizontal line marks the significant threshold IRR = 1

Fluralaner highly increased the risk of sand fly death (Table 2). The incidence rate ratio (IRR) was 32.9 (95% CI: 4–263), 76 (95% CI: 8–705), 95.8 (95% CI: 9–1029) and 10.6 (95% CI: 1.43–79) on Days 3, 17, 31 and 45, respectively (Table 2). The sand fly killing effect of fluralaner decreased after Day 31 post-treatment and on Day 73 the effect was not significant (Fig 3).

**Table 2:** Negative binomial mixed model results including treatment group, experimental day and their interaction as fixed effects and dog as random effect. The incidence rate ratio (IRR) represents the increase in the rate of incidence compared with control per day

Explanatory variable^a^	IRR^b^	95% CI	*P*-value
Day -7	0.75	0.09–4.85	0.77
Day 3	32.87	4.09–263	0.001**
Day 17	76.06	8.20–705	0.0002**
Day 31	95.85	8.92–1029	0.0002**
Day 45	10.66	1.43–79	0.02*
Day 73	2.50	0.34–18	0.36

^a^The explanatory variables used control group at each corresponding day after treatment as baseline comparison (Pr(>|z|))

^b^Incidence rate ratio, as the ratio between the incidence rate in control group and the incidence rate for each treatment group, at the corresponding experimental day after treatment

*Significance level at α = 0.05

**Significance level at α = 0.001

## Discussion

One oral dose of Bravecto® showed efficacies > 94% at killing sand flies for 31 days and > 75 % for 45 days post-treatment. The sand fly risk of death 24 h after feeding increases more than 10 times when feeding from a treated dog and this effect was maintained for 45 days after treatment.

The efficacy of fluralaner administered to dogs at killing sand flies has already been demonstrated in a membrane-feeding study [6]. However, the mentioned study reported lower mortalities in the fluralaner group (42–80%) and higher mortalities in the control group (35–60%). These discrepancies with our results could be due to the manipulation of the blood samples (freeze-thaw) made during the membrane-feeding study [30]. A recent laboratory study evaluated the IC_50_ (concentration at which 50% of the sand flies died 24 h after feeding) of fluralaner for two sand fly species, *Lutzomyia longipalpis* (vector of *L. infantum* in Latin America) and *P. argentipes* (vector of *L. donovani* in the Indian subcontinent). *Lutzomyia longipalpis* required twice the dose of fluralaner [655 ng/ml (95% CI: 537–796)] to kill 50% of the sand flies compared to *P. argentipes* [318 ng/ml (95% CI: 278–364)]. Bravecto® demonstrated a plasma concentration of fluralaner maintained above 650 ng/ml for 40 days and above 350 ng/ml, for 56 days post-treatment [1, 3, 31]. Our study found *P. patatasi* mortality above 75% for 45 days post-treatment. *Phlebotomus papatasi* has shown to be as susceptible to fluralaner as *P. argentipes* and more susceptible than *L. longipalpis*. In the Old World, *P. perniciosus* is the main vector of *L. infantum*, thus the experiment should have ideally been performed using a colony of *P. perniciosus*. However, the trial required a total of 1800 females and unfortunately the colony of *P. perniciosus* in the University of Zaragoza could not produce this number of sand flies; as such we had to use *P. papatasi* instead. Based on a previous study we expect similar insecticide sensitivity between *P. papatasi* and P*. perniciosus* [32]. Because a higher concentration of fluralaner is needed to control *L. longipalpis*, a shorter period of action of Bravecto® could be expected in this sand fly species. Additionally, Bravecto® administered to dogs also induced 100% triatomine mortality for 51 days [21].

## Conclusions

A single chewable tablet of fluralaner was effective against sand flies feeding on treated dogs for 45 days (efficacy > 75%). The community-wide use of fluralaner and other isoxazoline drugs registered for dogs such as afoxolaner or saloraner should be evaluated as a ZVL control strategy in endemic areas.

## Additional file

Additional file 1: **Table S1.** Sand fly mortality, percentage, and 95% CI calculated using the Kaplan-Meier method from the mortality observed 24, 48, 72, 96 and 120 h after direct blood-feeding on dogs by treatment and sampling day. **Table S2.** Individual dog sand fly mortality and percentage observed 24 h after direct blood-feeding by treatment and sampling day. (DOCX 40 kb)

## Abbreviations

CI: Confidence interval
IRR: Incidence rate ratio
ZVL: Zoonotic visceral leishmaniasis
