# Supplementary material for: A randomized, blinded, controlled trial to assess sand fly mortality of fluralaner administered orally in dogs
Source: Parasit Vectors. 2018 Dec 5;11:627. doi: 10.1186/s13071-018-3231-8 (PMC6282346; doi:10.1186/s13071-018-3231-8)
Supplement: Supplementary file 1 — Table S1. Sand fly mortality, percentage, and 95% CI calculated using the Kaplan-Meier method from the mortality observed 24, 48, 72, 96 and 120 h after direct blood-feeding on dogs by treatment and sampling day. Table S2. Individual dog sand fly mortality and percentage observed 24 h after direct blood-feeding by treatment and sampling day. (DOCX 40 kb) [file 13071_2018_3231_MOESM1_ESM.docx]

**Table S1** Sand fly mortality, percentage, and its 95% CI calculated using Kaplan Meier method from the mortality observed at 24, 48, 72, 96 and 120 hours after direct blood feeding on dogs by treatment and sampling day

| Group | Hours after direct feeding |  |  | Days after treatment | | | | | |
| --- | --- | --- | --- | --- | --- | --- | --- | --- | --- |
|  |  |  |  | Day -7 | Day 3 | Day 17 | Day 31 | Day 45 | Day 73 |
| Sand flies |  | n |  | 138 | 147 | 150 | 148 | 141 | 122 |
| Control | 24 | ^1^Deaths (%) |  | 3 (2) | 6 (4) | 3 (2) | 2 (1) | 13 (10) | 25 (20) |
|  |  | ^2^95% CI |  | (0, 5) | (0, 7) | (0, 4) | (0, 3) | (5, 15) | (13, 28) |
|  | 48 | Deaths (%) |  | 9 (9) | 35 (28) | 9 (8) | 12 (10) | 19 (23) | 36 (50) |
|  |  | 95% CI |  | (4, 14) | (21, 35) | (4, 13) | (5, 14) | (16, 30) | (41, 59) |
|  | 72 | Deaths (%) |  | 5 (12) | 26 (46) | 14 (17) | 14 (19) | 12 (32) | 14 (62) |
|  |  | 95% CI |  | (7, 18) | (38, 54) | (11, 23) | (13, 25) | (24, 39) | (53, 70) |
|  | 96 | Deaths (%) |  | 13 (22) | 29 (65) | 19 (30) | 12 (27) | 10 (38) | 6 (67) |
|  |  | 95% CI |  | (15, 29) | (58, 73) | (23, 37) | (20, 34) | (30, 46) | (58, 75) |
|  | 120 | Deaths (%) |  | 8 (28) | 2 (67) | 18 (42) | 13 (36) | 2 (40) | 5 (70) |
|  |  | 95% CI |  | (20, 35) | (59, 74) | (34, 50) | (28, 44) | (32, 48) | (62, 79) |
| Sand flies |  | n |  | 123 | 151 | 146 | 159 | 148 | 182 |
| Fluralaner | 24 | Deaths (%) |  | 2 (2) | 151 (100) | 143 (98) | 150 (95) | 114 (77) | 75 (42) |
|  |  | 95% CI |  | (0, 4) |  | (96, 100) | (91, 98) | (72 ,82) | (35, 49) |
|  | 48 | Deaths (%) |  | 9 (9) |  | 1 (99) | 9 (100) | (76, 88) | 58 (73) |
|  |  | 95% CI |  | (4, 14) |  | (97, 100) | NA | 2 (83) | (67, 80) |
|  | 72 | Deaths (%) |  | 10 (17) |  | 2 (100) | NA | (77, 90) | 29 (89) |
|  |  | 95% CI |  | (10, 24) |  | ^1^NA | NA | 25 (100) | (85, 94) |
|  | 96 | Deaths (%) |  | 13 (28) |  | NA | NA | NA | 6 (93) |
|  |  | 95% CI |  | (20, 36) |  | NA | NA | NA | (89, 97) |
|  | 120 | Deaths (%) |  | 9 (35) |  | NA | NA | NA | 6 (95) |
|  |  | 95% CI |  | (27, 43) |  | NA | NA | NA | (90, 99) |

^1^NA samples showed 100% deaths in the observation before

**Table S2** Individual dog sand fly mortality and its percentage observed at 24 hours after direct blood feeding by treatment and sampling day

|  |  |  | Day -7 | | Day 3 | | Day 17 | | Day 31 | | Day 45 | | Day 73 | |
| --- | --- | --- | --- | --- | --- | --- | --- | --- | --- | --- | --- | --- | --- | --- |
| Dod_ID | Gender | Treatment | n | deaths (%) | n | deaths (%) | n | deaths (%) | n | deaths (%) | n | deaths (%) | n | deaths (%) |
| 1343 | Female | Control | 30 | 1 (3) | 30 | 2 (7) | 32 | 0 | 34 | 0 | 26 | 0 | 30 | 8 (27) |
| 1349 | Male | Control | 31 | 1 (3) | 27 | 1 (4) | 26 | 0 | 30 | 1 (3) | 29 | 2 (7) | 31 | 11 (36) |
| 1352 | Female | Control | 22 | 0 | 30 | 1 (3) | 31 | 2 (6) | 33 | 1 (3) | 24 | 10 (42) | 31 | 9 (29) |
| 1357 | Male | Control | 30 | 1 (3) | 30 | 0 | 31 | 0 | 20 | 0 | 31 | 0 | 30 | 3 (10) |
| 1451 | Female | Control | 25 | 0 | 30 | 2 (7) | 30 | 1 (3) | 31 | 0 | 31 | 1 (3) | 31 | 3 (10) |
| 1333 | Female | Fluralaner | 25 | 0 | 31 | 31 (100) | 30 | 30 (100) | 33 | 32 (99) | 26 | 24 (98) | 60 | 32 (54) |
| 1377 | Male | Fluralaner | 27 | 1 (4) | 30 | 30 (100) | 31 | 31 (100) | 32 | 32 (100) | 32 | 32 (100) | 31 | 5 (16) |
| 1485 | Male | Fluralaner | 28 | 0 | 30 | 30 (100) | 24 | 21 (88) | 33 | 33 (100) | 34 | 26 (77) | 30 | 5 (17) |
| 5503 | Male | Fluralaner | 28 | 1 (4) | 30 | 30 (100) | 30 | 30 (100) | 30 | 27 (90) | 25 | 20 (80) | 31 | 6 (19) |
| 5599 | Female | Fluralaner | 15 | 0 | 30 | 30 (100) | 31 | 31 (100) | 31 | 26 (84) | 31 | 31 (100) | 30 | 27 (90) |
